# *E. coli Nissle 1917* ameliorates mitochondrial injury of granulosa cells in polycystic ovary syndrome through promoting gut immune factor IL-22 *via* gut microbiota and microbial metabolism

**DOI:** 10.3389/fimmu.2023.1137089

**Published:** 2023-05-19

**Authors:** Man Luo, Yuanyuan Chen, Xiangyang Pan, Hongmei Chen, Lang Fan, Yi Wen

**Affiliations:** Reproductive Medicine Center, Hunan Provincial Maternal and Child Health Hospital (Hunan Provincial Reproductive Medicine Institution), Changsha, China

**Keywords:** *E. coli Nissle 1917*, IL-22, polycystic ovary syndrome, mitochondrial injury, microbial metabolism

## Abstract

**Objective:**

Gut microbiota and its metabolites have regulatory effects on PCOS related ovarian dysfunction and insulin resistance. *Escherichia coli Nissle 1917* (EcN) is a genetically controlled probiotic with an excellent human safety record for improving gut microbiome metabolic disorders and immune system disorders. Here we focused to explore the application and effect of probiotic EcN on the gut microbiota-metabolism-IL-22-mitochondrial damage axis in PCOS.

**Methods:**

PCOS mice were constructed with dehydroepiandrosterone (DHEA) and treated with EcN, FMT or IL-22 inhibitors. Clinically control and PCOS subjects were included for further analysis. Serum and follicular fluid supernatant levels of sex hormones, insulin, glucose, cholesterol, and inflammatory factors were detected by ELISA and biochemical reagents. The pathological changes of ovarian tissues were observed by HE staining. The JC-1 level and COX4 gene expression in granulosa cells was detected by ELISA and RT-qPCR. The expressions of progesterone receptor A (PR-A), LC3II/I, Beclin1, p62 and CytC were detected by western blot. The number of autophagosomes in granulosa cells was observed by electron microscopy. 16S rRNA and LC-MS/MS were used to analyze the changes of gut microbiota and metabolism.

**Results:**

EcN promoted the recovery of sex hormone levels and ovarian tissue morphology, promoted the expression of IL-22, COX4 and PR-A in granulosa cells, and inhibited mitophagy in PCOS mice. EcN decreased the number of gut microbiota, and significantly increased the abundance of *Adlercreutzia*, *Allobaculum*, *Escherichia-Shigella* and *Ileibacterium* in PCOS mice. EcN improved metabolic disorders in PCOS mice by improving Amino sugar and nucleotide sugar metabolism pathways. IL-22 was positively associated with *Ileibacterium*, *Adlercreutzia* and Progesterone, negatively associated with *RF39*, Luteinizing hormone, Testosterone, N−Acetylglucosamin, L−Fucose and N−Acetylmannosamin. FMT reconfirmed that EcN ameliorated mitochondrial damage in granulosa cells of PCOS mice by gut microbiota, but this process was blocked by IL-22 inhibitor. Clinical trials have further demonstrated reduced IL-22 levels and mitochondrial damage in granulosa cells in PCOS patients.

**Conclusion:**

EcN improved IL-22 level and mitochondrial damage of granulosa cells in PCOS mice by promoting the recovery of sex hormone levels and ovarian tissue morphology, inhibiting the amount of gut microbiota, and promoting amino sugar and nucleotide sugar metabolism.

## 1 Introduction

Currently, women with PCOS have dysregulated expression of endometrial hormone receptors and insulin resistance, resulting in impaired glucose transport, as well as chronic low-grade inflammation, immune dysfunction, abnormal expression of endometrial genes, and mitochondrial dysfunction (1, 2). Type 1 diabetes associated with glucose homeostasis occurs frequently in PCOS when the ovaries and adrenal glands are exposed to excessive insulin concentrations (3). The regulation of glucose homeostasis and insulin resistance was one of the therapeutic approaches to improve the endocrine disorder of PCOS (4). In addition, dysregulation of the gut microbiota has been proposed as a potential causative factor in the development of PCOS (5). Studies have shown that intestinal microbial dysregulation in PCOS is associated with the clinical phenotype of the disease and leads to insulin resistance (6, 7). The intake of probiotics developed based on gut microbes may improve glucose homeostasis associated with PCOS (8). Therefore, probiotic could be as a dietary therapy for gut microbiota disorders and endocrine disorder of PCOS.

Engineered microbes, such as reasonable engineered variants of *E. coli strain Nissle*, are rapidly being developed to deliver therapeutic modalities to disease sites (9). Among Gram-negative microorganisms with probiotic properties, *E. coli strain Nissle 1917* (EcN) is probably the most intensively studied strain today (10). EcN can alter the glycemic response to glucose infusion, but this effect is not mediated by direct glucose uptake and may involve other agents (11). EcN reduced serum leptin and insulin resistance and increased α-diversity of gut microbiota in obese C57BL/6J mice (12). The protective effect of engineered EcN on dextran sodium sulfate (DSS) -induced colitis in mice was enhanced, which was related to mucosal healing and immune regulation (13). However, the role of EcN in PCOS is still unknown and deserves further study.

Interleukin-22 (IL-22) belongs to the IL-10 family and is an α-helical cytokine specifically produced by many lymphocytes such as Th1, Th17, Th22, ILC, CD4+, and CD8+ T cells (14). Intestinal microbiota and intestinal immune factor IL-22 play important roles in the pathogenesis of PCOS. Studies have shown that IL-22-associated Browning of white adipose tissue modulates insulin sensitivity and ovarian function in PCOS (15). Bacteroides down-regulate bile acid metabolism pathway, resulting in intestinal lymphocytes unable to respond to secondary bile acid stimulation, resulting in insufficient secretion of anti-inflammatory factor IL-22, leading to PCOS (16). EcN flagellin mediates symbiotic properties through stronger TLR5 activation, resulting in IL-22-mediated protection against DSS-induced colitis in mice (17). Whether EcN regulates the intestinal immune factor IL-22 to restore gut microbiota homeostasis and alleviate PCOS remains unclear.

In the ovary, macro-autophagy/autophagy plays a key role in directing the chain of events from oocyte origin to fertilization, and defects in autophagy of follicle cells at different stages of follicles were observed in PCOS ovaries (18). Granulosa cells play an important role in oocyte development and maturation (19). Mitochondrial damage in granulosa cells is related to the pathogenesis of PCOS (20, 21). Il-22 has been shown to repair mitochondrial damage by down-regulating uncoupling protein 2 in insulin-secreting rat cells (22). A microbiota-derived metabolite of the flavonoid quercetin mediates mitochondrial damage in colon cancer cells (23). However, whether up-regulation of IL-22/gut microbiota mediates mitochondrial damage in granulosa cells in PCOS remains unclear. Therefore, in this study, PCOS mice were constructed and treated with EcN, FMT and IL-22 inhibitors, and clinical subjects were collected for IL-22 levels and mitochondrial damage detection. In order to explore the mechanism of EcN and IL-22 in intestinal microbial metabolism dysregulation and ovarian granulosa cell autophagy injury related to PCOS disease, and provide new insights for the treatment of PCOS.

## 2 Materials and methods

### 2.1 PCOS mice with EcN intervention

The experiments were approved by the Ethics Committee of Hunan Provincial Maternal and Child Health Hospital (2022037). A total of 30 female C57BL/6 mice (21 days old, 8~10 g) were purchased from Lake Jingda Laboratory Animal Center, Hunan. The mice were raised at a temperature of 18 to 29°C, a relative humidity of 40 to 70, and 12/12h of light. Mice were free to eat and drink. The standard feed and drinking water for mice were autoclaved. Mice were fed adaptively for one week. Mice were divided into Control group, DHEA-induced PCOS group and *E. coli Nissle 1917* (EcN) group, 10 mice/group. Mice in DHEA-induced PCOS group and EcN group were subcutaneously injected with DHEA (6 mg/100 g body weight, HY-14650, MCE, USA) once a day for 3 weeks to construct the PCOS model. DHEA was dissolved with sesame oil (0.09 mL) and ethanol (95%, 0.01 mL) at room temperature (24). In making mold period, the estrus cycle (proestrus, estrus, metestrus, and diestrus) was examined by vaginal smears for 14 days. When the estrous cycle was disturbed, the PCOS mouse model was successfully established (21, 24). After modeling, mice in DHEA-induced PCOS group and EcN groups were treated by gavage with 0.9% NaCl and EcN (10^9^ CFU/0.2 mL, dissolved in 0.9% NaCl, HG-VDH1186, HonorGene) for 4 weeks (25). During the experiment, mice in Control group were injected with sesame oil (0.09 mL) and ethanol (95%, 0.01 mL) for 3 weeks, subsequently by gavage with 0.9% NaCl for 4 weeks as control. All the drugs intervention were performed at 8 am per day. The experimental flow chart was showed in the **Supplementary Figure 1**. At the end of the experiment, mice were euthanized with 150 mg/kg pentobarbital sodium (I.P). Peripheral blood, ovary, granulosa cells, and fecal samples (Terminal colorectal contents, 10 samples/group) were collected for subsequent detection. Granulosa cell (GC) was released and collected by killing mice 44 h after intraperitoneal injection of 5 IU PMSG and then puncturing antral follicles with a 26.5-g needle (26). Fecal samples (Terminal colorectal contents, 10 samples/group) were collected from the terminal colorectal contents and stored in a 1.5 mL sterile enzyme free tube at -80°C.

### 2.2 Detection of estrus cycle in mice

Due to the sexual maturation of mice at 6-7 weeks of age, we conducted estrus cycle testing for 14 consecutive days after a week of modeling intervention. The estrus cycle (proestrus, estrus, metestrus, and diestrus) were examined by vaginal smears (21, 24). Briefly, the mice were kept in the left hand to be tested, and the right hand was used to absorb a small amount of normal saline preheated at 37°C with a sterilized dropper. The dropper head was put into the vaginal opening of the mouse, gently and repeatedly squeeze the dropper rubber head 3~5 times to moisten the mouse’s vagina. Then, 1 drop of normal saline was put in the dropper onto a clean slide. The droplet was laid flat on the slide with the dropper head, and then placed in a ventilated dry place at room temperature to dry thoroughly. The sample area was covered with 95% alcohol and fixed for 5~8 min. The samples were stained with hematoxylin dyeing solution for 20~30s. The samples were differentiated by drops of 1% hydrochloric acid ethanol solution, and immediately stained with eosin solution for 1 min after 5s. Then the sections were washed with running water to remove excess staining solution and observed under a microscope (BA210T, Motic). Subsequently, estrus status was determined by the characteristics of the vulva and the epithelial cells of the vaginal exfoliation at different stages of the estrus cycle (**Supplementary Figures 2** and **3**). The criteria are as follows: 1) A large number of nuclear epithelial cells appear to be in proestrus; 2) Squamous epithelial cells accounted for most cell types during estrus; 3) Nuclear epithelial cells, squamous epithelial cells and white blood cells can be seen in metestrus. 4) Almost all cells in diestrus are white blood cells.

### 2.3 Fecal microbiota transplantation (FMT)

A total of 10 female C57BL/6 mice (21 days old) were purchased from Lake Jingda Laboratory Animal Center, Hunan. All mice were randomly divided into two groups: DHEA-induced PCOS -FMT group and E. coli Nissle 1917-FMT group, 5 mice/group (**Supplementary Figure 1**). Fecal samples (Terminal colorectal contents) were collected from donor mice in the above groups (DHEA-induced PCOS group and E. coli Nissle 1917 group). Fecal samples (Terminal colorectal contents, 200 mg) were resuspended in 1mL of sterile saline for subsequent FMT experiments (27). The solution was mixed vigorously for 10 s, then centrifuged at 800×g for 3 min to collect the supernatant and used as graft material. To prevent changes in bacterial composition, fresh graft material was prepared on the day of transplantation within 10 min before tube feeding. Before the FMT, mice were given orally an antibiotic cocktail containing ampicillin (0.1 mg/mL), streptomycin (0.5 mg/mL) and colistin (0.1 mg/mL) to ablate gut microbiota for 4 days, normal drinking water for 3 days (27). Mice in DHEA-induced PCOS-FMT group and E. coli Nissle 1917-FMT group were given 0.1 mL fecal supernatant of mice in DHEA-induced PCOS group and E. coli Nissle 1917 group by gavage every day for 7 days (16, 27). All the drugs intervention were performed at 8 am per day. At the end of the experiment, mice were euthanized with 150 mg/kg pentobarbital sodium (I.P). Peripheral blood, ovary and granulosa cell specimens were collected for subsequent detection.

### 2.4 Intervention of IL-22 in FMT-PCOS mice

A total of 25 female C57BL/6 mice (21 days old) were purchased from Lake Jingda Laboratory Animal Center, Hunan. All mice were randomly divided into 5 groups: Control group, DHEA-induced PCOS group, α-IL-22 group, E. coli Nissle 1917-FMT group, α-IL-22 +E. coli Nissle 1917-FMT group, 5 mice/group (**Supplementary Figure 1**). Except for Control group, the other groups established the PCOS mouse model according to the above methods. After 21 days of modeling in the α-IL-22 group, mice were given a single intraperitoneal (I.P) dose of 400 µg α-IL-22 (IL22JOP, Thermo Fisher, USA) and gavage of 0.1mL sterile saline (28). After 21 days of modeling, mice in the E. coli Nissle 1917-FMT group were given 0.1mL fecal supernatant of mice in the E. coli Nissle 1917 group daily for 7 days. Mice in the α-IL-22+E. coli Nissle 1917-FMT group were given α-IL-22 and fecal supernatant interventions for 7 days. All the mice were treated with 400 µg of mouse IgG2aκ (11-4724-42, Thermo Fisher, USA) and 0.1 mL of sterile saline intragastric for 7 days as control. All the drugs intervention were performed at 8 am per day. At the end of the experiment, mice were euthanized with 150 mg/kg pentobarbital sodium (I.P). Peripheral blood, ovary and granulosa cell specimens were collected for subsequent detection.

### 2.5 Clinical trials and therapeutic interventions

All subjects were recruited from Hunan Provincial Maternal and Child Health Hospital and were aged 22 to 38 years. All subjects were divided according to their diagnosis into women presenting with male azoospermia or fallopian tube obstruction (Control, n=15) and newly diagnosed patients with PCOS (n=15) (**Table 1**). Other causes of hyperandrogenemia or ovulation dysfunction (Cushing’s syndrome, 21-hydroxylase deficiency, thyroid disease, androgen-secreting neoplasms, congenital adrenal hyperplasia, and hyperprolactinemia) were excluded in subjects with PCOS. According to the 2003 Rotterdam criteria (16), at least two of the following are required for diagnosis: (1) hypo-ovulation and/or anovulation; (2) Clinical and/or biochemical signs of hyperandrogenism; (3) Polycystic ovary. The control group was from subjects with normal menstrual cycle, ovarian morphology, and hormone levels. All PCOS patients and controls in the above group were in the first IVF cycle, and fasting peripheral blood was collected at 8~10 am for detection after standardized intervention.

**Table 1:** Baseline characteristics of participants.

Variables	Control	PCOS	*P* value
Participants, *n*	15	15	—
Age, years	26.50 ± 6.88	31.13 ± 6.04	0.0602
BMI, kg/m^2^	23.14 ± 3.32	21.25 ± 2.78	0.1021

### 2.6 Collection and culture of follicular fluid and granulosa cells

The staff of the sample bank will standardize and collect the follicular fluid produced in the process of assisted reproductive technology treatment of the included patients. At the first puncture of each ovary from which the egg was retrieved, the follicular fluid samples were collected from a single large follicle and centrifuged to remove cells for subsequent analysis. The tops of cell precipitates were collected from concomitant follicular fluid samples of PCOS patients, which were aspirated and washed in PBS. Then, the cell precipitates were resuspended in PBS and cultured in Ficoll (LTS1077, TBD Science) to layer the solution and separate from red blood cells through centrifugation. The cell layer at the Ficoll/PBS interface were aspirated and washed with PBS to remove residual Ficoll. The final granulosa cells were precipitated in PMI-1640 medium supplemented with 10% fetal bovine serum and 1% penicillin-streptomycin (5000 U/mL) at 37°C in a moist atmosphere of 5% CO_2_ and used for subsequent assays.

### 2.7 ELISA

The blood and follicular fluid samples were placed at room temperature for 2 h and centrifuged at 1000g for 15 min at 2-8°C to obtain serum and follicular fluid supernatant. Then, they were stored at -80°C for detection. Serum and follicular fluid supernatants were used to measure the progesterone (P, CSB-E05104m, CUSABIO), Luteinizing Hormone (LH, CEA441Mu, CEA441Hu, cloud-clone corp), Testosterone (T, CEA458Ge, cloud-clone corp), Follicle Stimulating Hormone (FSH, CEA830Mu, SEA830Hu, cloud-clone corp), IL-22 (KE10041, KE00008, Proteintech), insulin (INS, CSB-E05069h, CUSABIO), Dehydroepiandrosterone Sulfate (DHEA-S, ab108669, abcam), Androstenedione (ASD, CEA456Ge, cloud-clone corp), Estradiol (E2, CEA461Ge, cloud-clone corp), sex hormone binding globulin (SHBG, SEA396Hu, cloud-clone corp), IL-6 (KE00118, Proteintech), IL-1β (DLB50, Bio-Techne China Co., Ltd) with a microplate reader (MB-530, HEALES).

### 2.8 Biochemical tests

The levels of triglyceride (TG, A110-1-1, NJJCBI), total cholesterol (T-CHO, A111-1-1, NJJCBI), high-density lipoprotein cholesterol (HDL-C, A112-1-1, NJJCBI), low-density lipoprotein cholesterol (LDL-C, A113-1-1, NJJCBI) and glucose (Glu, F006-1-1, NJJCBI) in serum and follicular fluid supernatant were detected by GPO-PAP, microplate, and peroxidase methods, respectively.

### 2.9 HE staining

Ovarian tissue was fixed, embedded, and sectioned. Sections were deparaffinized to water by xylene and graded ethanol. Sections were stained with hematoxylin and eosin sequentially. Sections were dehydrated with graded alcohol (95-100%) and observed by a microscope (BA210T, Motic).

### 2.10 RT-qPCR

Granulosa cells were collected and total RNA was extracted by TRIzol reagent (Thermo, USA). The cDNA was synthesized using a Kit (HiFiScript cDNA Synthesis Kit, CWBio, China). The primer sequence was H-COX4 (146 bp): F-AGGCCCAAGGAGAGAAGCTA, R-AGGCCCATCAACCTTACACG. H-GAPDH (100 bp): F-TCCTGCCCTTTGAGTTTGATGATGCT, R-ACTATGCCACCCCAGGAATGCTT. M-COX4 (149 bp): F-TCCCCACTTACGCTGATCG, R-GATGCGGTACAACTGAACTTTCT and M-GAPDH (122 bp), F-GCGACTTCAACAGCAACTCCC, R-CACCCTGTTGCTGTAGCCGTA. The relative mRNA expression levels of COX4 were analyzed by UltraSYBR Mixture (CW2601, CWBio, China) and 2^−ΔΔCT^ method.

### 2.11 Western blot

The cells were washed with ice-pre-cooled PBS and added with 200 uL RIPA lysate (AWB0136, Abiowell). The total protein was extracted after ultrasonic crushing for 1.5 min. Protein concentrations were determined using the BCA method. Protein samples (200 μg) were separated by SDS-PAGE (12%, AWT0047, Abiowell). Then, proteins were transferred to a polyvinylidene difluoride membrane, which was activated with methanol and blocked with skim milk powder (5%, AWB0004, Abiowell). Membrane was incubated with antibodies, which were included anti-progesterone receptor A (PR-A, ab32085, 1:5000, Abcam, UK), anti-LC3II/I (#43566, 1:1000, CST, USA), anti-Beclin1 (11306-1-AP, 1:1000, Proteintech, USA), anti-p62 (18420-1AP, 1:4000, Proteintech, USA), anti-CytC (10993-1-AP, 1:4000, Proteintech, USA) and anti-β-actin (66009-1-Ig, 1:5000, Proteintech, USA). Then, it was incubated with secondary anti-IgG (SA00001-1/2, 1:6000/5000, Proteintech, USA) at 37°C for 90 min. ECL Plus hypersensitive luminescence solution (AWB0005, Abiowell) was used for visualization, and software (ChemiScope6100, CLINX) was used for imaging analysis.

### 2.12 Mitochondrial membrane potential (MMP)

According to the manufacturer’s instructions, the levels of MMP (AWC0128a, Abiowell) was detected by JC-1 method. In brief, cells were re-suspended in 0.5 mL cell culture medium and mixed with 0.5 mL JC-1 staining working medium. The cells were incubated in an incubator at 37°C for 20 min. Cell precipitates were collected by centrifugation. Cells were resuspended and washed twice with JC-1 staining buffer (1×). The cells were suspended with JC-1 staining buffer (1×) and analyzed by flow cytometry (A00-1-1102, Beckman). The MMP (%) = UR (red fluorescence)/LR (green fluorescence).

### 2.13 Transmission electron microscope (TEM)

Cells were fixed in 2.5% glutaraldehyde and 1% osmic acid (18456, TED PELLA INC) for 6-12 h and 1-2h, respectively. The cells were dehydrated using gradient ethanol (30 ~100%) and propylene oxide (M25514, MERYER). Subsequently, the cells were immersed in propylene oxide: epoxy resin (1:1) and pure epoxy resin for 1-2 h and 2-3 h for embedding, and oven baked for 60h. The embedded block was taken out and repaired, then ultrathin sections were cut and copper mesh was retrieved. The sections were stained for electron (lead and uranium). Finally, sections were observed with a transmission electron microscope (7700, Hitachi) and images were recorded with a digital camera (ER-B, AMT).

### 2.14 16S rRNA sequence

DNA was extracted by the kit (Tiangen, cat. #DP328-02) from fecal samples. PCR amplification and library construction were conducted by phusion enzyme (K1031, APExBIO) and the V3-V4 region primers (357F 5’- ACTCCTACGGRAGGCAGCAG-3’ and 806R 5’-GGACTACHVGGGTWTCTCATAT-3’) of the 16S rRNA gene. Illumina NovaseQ6000 PE250 was applied for mixed sequence to collect Raw Data. Qiime 2 (2020.2) was adopted to improve the data quality control, and calculate the Alpha diversity index, PCA analysis and relative abundance of microbiota. Each ASV/OTU sequence was annotated by referring to the SilvA-132-99 database, and the corresponding species information and abundance distribution were obtained. R software (Venn Diagram package) and Jvenn web page (http://www.bioinformatics.com.cn/static/others/jvenn/example.html) were used to analyze common and unique ASVs in groups. LDA Effect Size analysis (LefSe, https://github.com/SegataLab/lefse) was applied for evaluate the differential microbiota.

### 2.15 LC-MS/MS

LC-MS/MS analysis was performed by the UHPLC system (1290, Agilent Technologies). The MS raw data files were converted for analysis through the R package XCMS (version 3.2). MetaboAnalyst platform (https://www.metaboanalyst.ca/) was used for bioinformatics analysis. The Kyoto Encyclopedia of Genes and Genomes (KEGG, https://www.kegg.jp/) pathway database was used for metabolite function prediction.

### 2.16 Data statistics and analysis

Statistical analysis of the data in this study was performed using Graphpad Prism8.0 statistical software. The measurement data are expressed as the mean ± standard deviation (SD). First, the normality and homogeneity of variance are tested. The test conformed to the normal distribution and the variance was uniform. The unpaired t test was used between groups. The one-way ANOVA or the analysis of variance of repeated measurement data is used for the comparison among groups. Tukey’s performed *post hoc* tests. *P* < 0.05 indicated that the difference was statistically significant.

## 3 Results

### 3.1 EcN improved mitochondrial damage and promoted IL-22 expression in granulosa cells of PCOS mice

**Figure 1:**
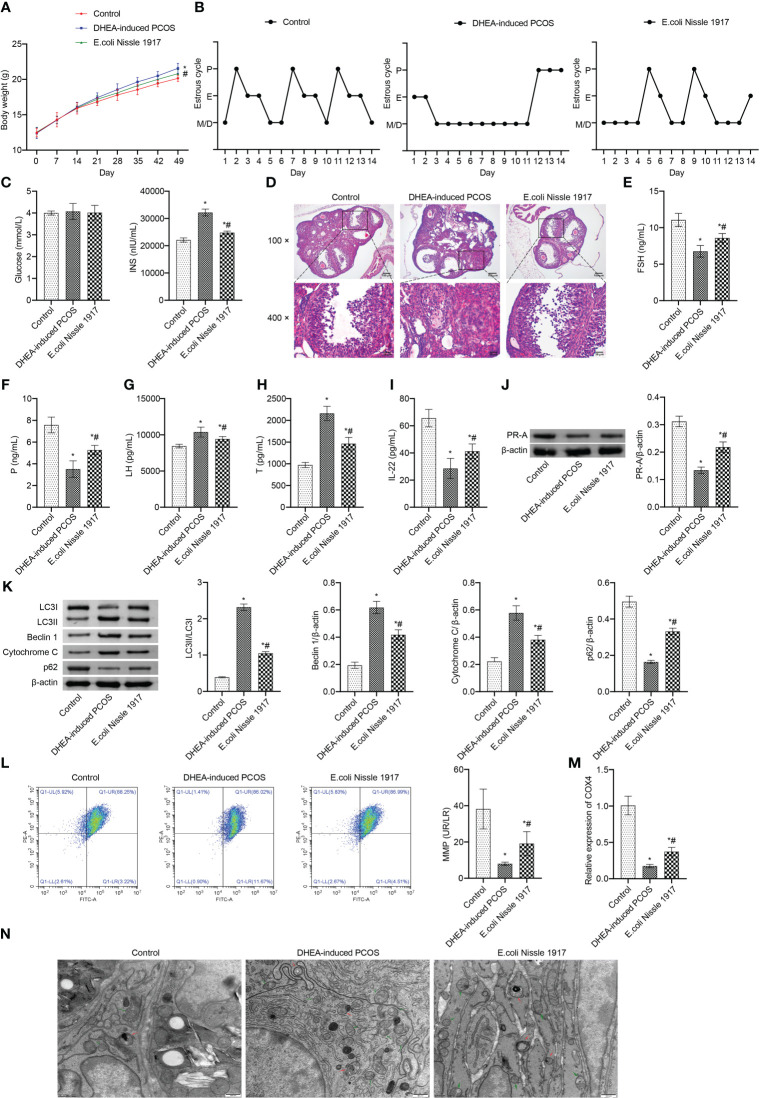
EcN improved mitochondrial damage and promoted IL-22 expression in granulosa cells of PCOS mice. **(A)** Body weight; **(B)** Estrus cycle determination; **(C)** Glucose and INS levels; **(D)** The morphological of ovary was analyzed by HE staining; **(E–I)** Serum levels of FSH, P, LH, T and IL-22 were detected by ELISA; **(J, K)** Western blot was used to detect the expression of PR-A, LC3II/I, Beclin 1, p62 and cytochrome C in ovarian tissues; **(L)** The MMP level in granulosa cells was detected by JC-1 method; **(M)** The expression of COX4 in granulosa cells was detected by RT-qPCR; **(N)** Mitochondrial damage in ovarian tissue was observed by electron microscopy (green arrow marked the damaged mitochondria, red arrow marked autophagosomes). *P<0.05 vs Control, #*P*<0.05 vs DHEA-induced PCOS. All data showed as mean ± SD.

From d14~d49, the body weight of mice in DHEA-induced PCOS group was higher than that in Control group, which was decreased after EcN intervention (**Figure 1A**). The estrus cycle detection showed that the Control group and E. coli Nissle 1917 group had normal cycles, while the DHEA-induced PCOS group had disrupted cycles (**Figure 1B**). The representative picture of the vaginal smears in Control group and DHEA-induced PCOS group (14 days) was shown in **Supplementary Figures 2** and **3**. There was no significant change of glucose level in mice of Control, DHEA-induced PCOS and EcN groups (**Figure 1C**). The INS level was rose in PCOS mice, which was down-regulated by EcN (**Figure 1C**). HE observation of ovarian morphology showed that the ovaries of the Control group contained follicles at different stages of development and several corpus luteum (**Figure 1D**). The characteristic polycystic ovary morphology was found in the ovaries of mice in the DHEA-induced PCOS group, with less corpus luteum, thinner granulosa cell layer, increased number of antral follicles and fewer primordial follicles (**Figure 1D**). EcN intervention resulted in relief of DHEA-induced PCOS symptoms (**Figure 1D**). The serum FSH and P levels were decreased and the serum Testo and LH levels were increased in PCOS mice (**Figures 1E–H**). EcN restored serum FSH and P levels and decreased Testo and LH levels in PCOS mice (**Figures 1E–H**). In addition, serum IL-22 levels were reduced in PCOS mice and were restored by EcN intervention (**Figure 1I**). The LC3II/I, Beclin 1 and Cytochrome C expression increased, while the expression of p62 and PR-A decreased in ovarian tissues of PCOS mice (**Figures 1J, K**). EcN inhibited the expression of LC3II/I, Beclin 1 and Cytochrome C and promoted the expression of p62 and PR-A in ovarian tissues of PCOS mice (**Figures 1J, K**). MMP level of PCOS mice was significantly decreased, which was reversed by EcN intervention (**Figure 1L**). The expression of COX4 in granulosa cells of PCOS mice was significantly decreased, and EcN intervention promoted the expression of COX4 in granulosa cells (**Figure 1M**). TEM showed that compared with Control group, there were many damaged mitochondria with the fracture, reduction or disappearance of ridge structure in the DHEA-induced PCOS group, which were engulfed by autophagosomes, indicating that autophagy was activated (**Figure 1N**). EcN intervention reduced the damaged mitochondria and autophagosomes in granulosa cells to inhibit the autophagy (**Figure 1N**). EcN improved mitochondrial damage and promoted IL-22 expression in granulosa cells of PCOS mice.

### 3.2 EcN remodeling of intestinal microbiota in PCOS mice

**Figure 2:**
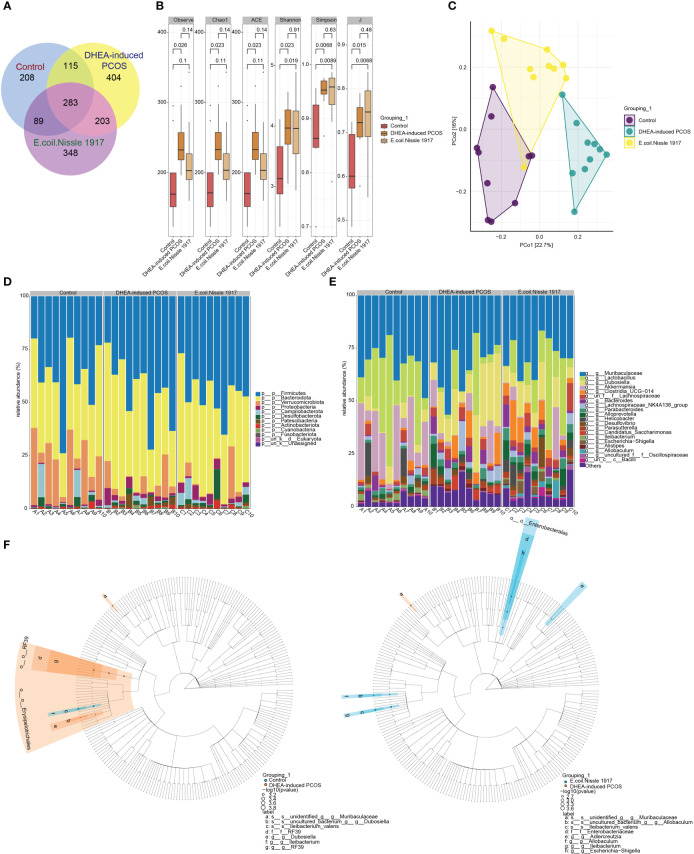
E. coli Nissle 1917 improved microbial diversity in PCOS mice. **(A)** Venn diagram showed the microbial quantity in different groups; **(B)** Alpha diversity index analysis; **(C)** PCoA analysis showed the diversity of sample community composition between groups; **(D, E)** Microbiota abundance at phylum level and genus level; **(F)** Difference microbiota was analyzed between Control group and DHEA-induced PCOS group, DHEA-induced PCOS group and E. coli Nissle 1917 group by Lefse.

The number of gut microbiota in PCOS mice was increased (**Figure 2A**). EcN treatment decreased the number of gut microbiota in PCOS mice (**Figure 2A**). The Alpha index of PCOS mice was significantly increased (**Figure 2B**). EcN treatment reduced the alpha index of PCOS mice, but there was no significant difference (**Figure 2B**). PcoA analysis showed that the samples of DHEA-induced PCOS group were far away from the Control group, while the samples of E. coli Nissle 1917 group were close to the Control group and there was crossover (**Figure 2C**). The microbiota at phylum level was mainly composed of Firmicutes, Bacteroidota, Verrucomicrobiota, Proteobacteria, Campilobacterota, Desulfobacterota, Patescibacteria, Actinobacteriota and Cyanobacteria (**Figure 2D**). The microbiota at genus level was mainly composed of *Muribaculaceae*, *Lactobacillus*, *Dubosiella*, *Akkermansia*, *Clostridia_UCG-014*, *Bacteroides*, *Lachnospiraceae_NK4A136_group*, *Parabacteroides*, *Alloprevotella*, *Helicobacter*, *Desulfovibrio*, *Parasutterella*, *Candidatus_Saccharimonas*, *Ileibacterium*, *Escherichia-Shigella*, *Alistipes* and *Allobaculum* (**Figure 2E**). The differences between groups showed that *Dubosiella*, *RF39* and *Ileibacterium* were significantly enriched in DHEA-induced PCOS group, while *Adlercreutzia*, *Allobaculum*, *Escherichia-Shigella* and *Ileibacterium* were significantly enriched in E. coli Nissle 1917 group (**Figure 2F**). These results demonstrated that EcN improved the microbial diversity and composition of PCOS mice.

### 3.3 EcN restored microbial metabolic homeostasis in PCOS mice

**Figure 3:**
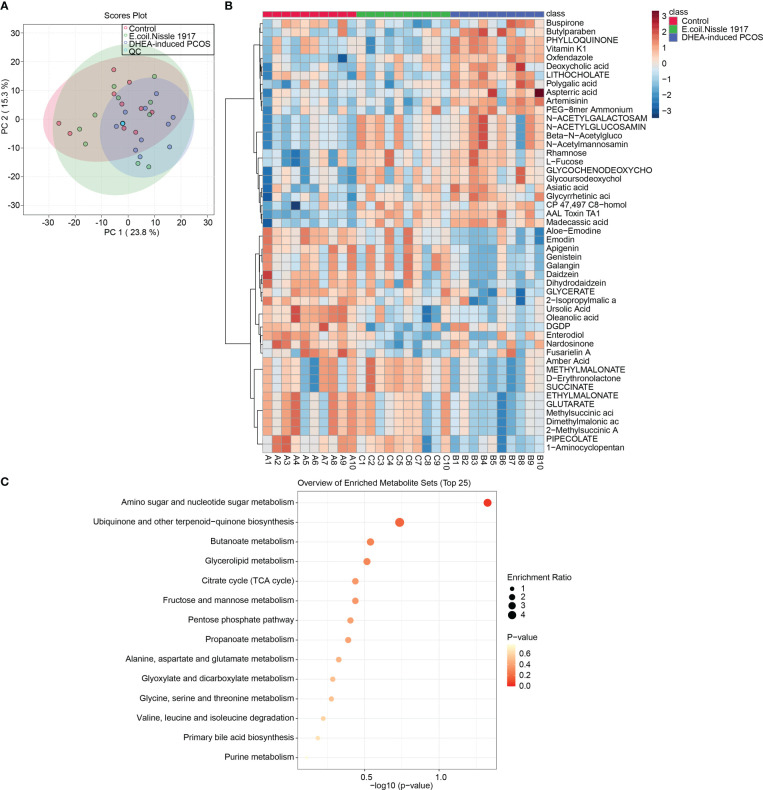
EcN improves metabolic characterization in PCOS mice. **(A)** PCA analysis; **(B)** Heatmap showed differential metabolite abundances; **(C)** KEGG predicted differential metabolite function.

PCA analysis showed that QC samples were clustered together, which proved that the instrument was stable (**Figure 3A**). The Heatmap showed the variation in abundance of differential metabolites across samples (**Figure 3B**). KEGG prediction showed that metabolites were enriched in the amino sugar and nucleotide sugar metabolism pathway (**Figure 3C**). All these proved that EcN has the most significant influence on amino sugar and nucleotide sugar metabolism pathway in PCOS mice.

### 3.4 IL-22 was correlated with the microbiota and metabolite

**Figure 4:**
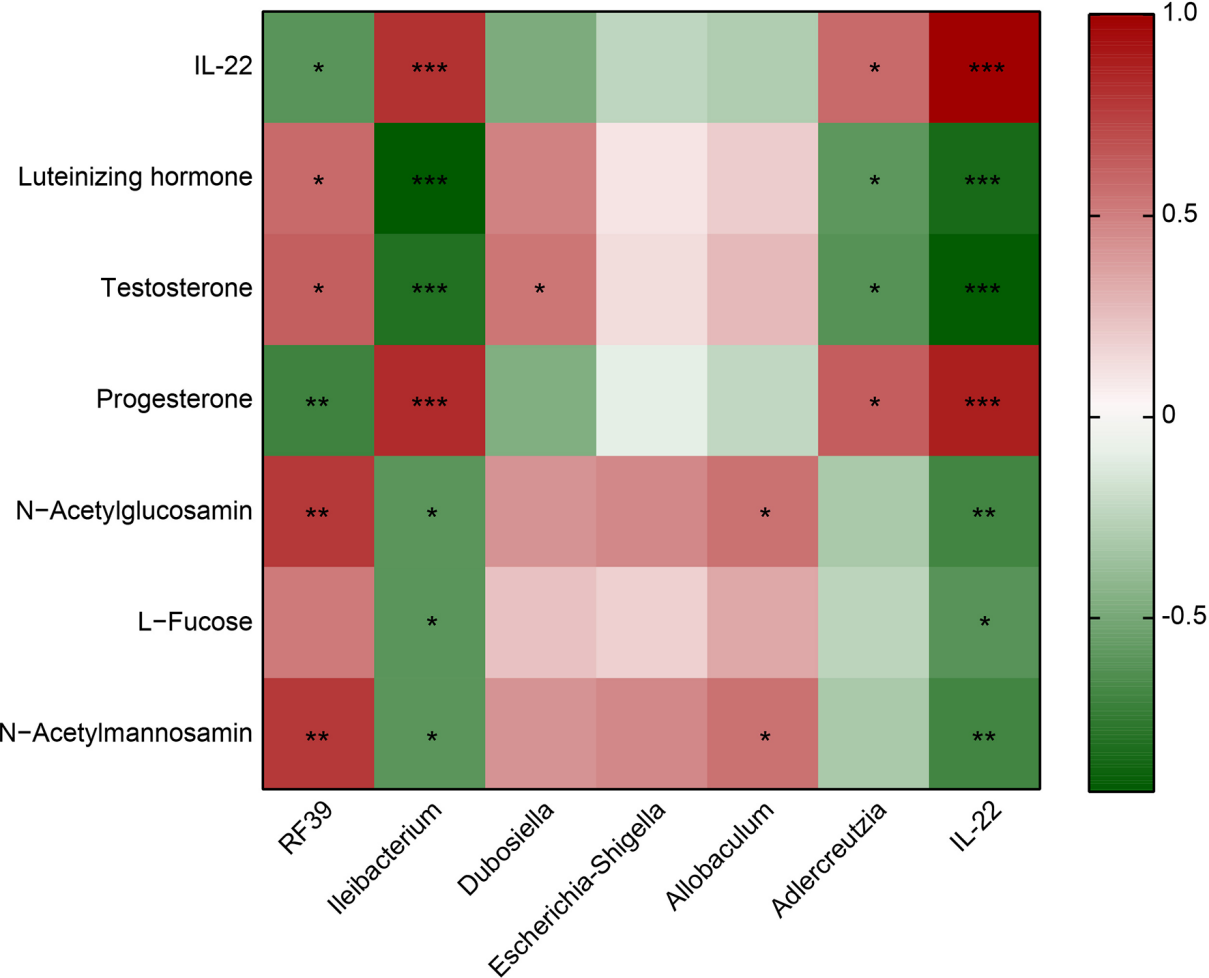
Heatmap showed the correlation between IL-22 and the microbiota or metabolite. Red represents positive correlation; green represents negative correlation. *P<0.05, **P<0.01, ***P<0.001.

*RF39* was positively correlated with Luteinizing hormone, Testosterone, N−Acetylglucosamin and N−Acetylmannosamin, negatively correlated with IL-22 and Progesterone (**Figure 4**). *Ileibacterium* was positively associated with IL-22 and Progesterone, negatively associated with Luteinizing hormone, Testosterone, N−Acetylglucosamin, L−Fucose and N−Acetylmannosamin (**Figure 4**). *Dubosiella* was positively correlated with Testosterone (**Figure 4**). *Allobaculum* was positively correlated with N−Acetylglucosamin and N−Acetylmannosamin (**Figure 4**). *Adlercreutzia* was positively associated with IL-22 and Progesterone, negatively associated with Luteinizing hormone and Testosterone (**Figure 4**). IL-22 was positively associated with Progesterone, negatively associated with Luteinizing hormone, Testosterone, N−Acetylglucosamin, L−Fucose and N−Acetylmannosamin (**Figure 4**). These results proved that IL-22 was positively associated with *Ileibacterium*, *Adlercreutzia* and Progesterone, negatively associated with *RF39*, Luteinizing hormone, Testosterone, N−Acetylglucosamin, L−Fucose and N−Acetylmannosamin.

### 3.5 E. coli Nissle 1917-FMT ameliorated mitochondrial damage in granulosa cells of PCOS mice

**Figure 5:**
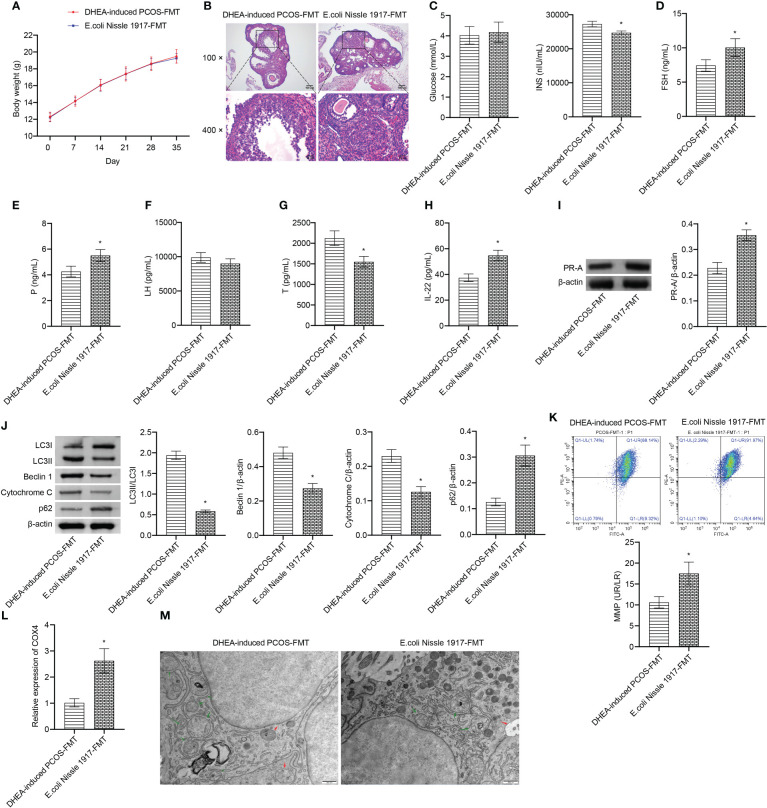
E. coli Nissle 1917-FMT ameliorated mitochondrial damage in PCOS mice. **(A)** Body weight; **(B)** HE staining; **(C)** Glucose and INS levels; **(D–H)** Serum levels of FSH, P, LH, Testo and IL-22 were detected by Elisa; **(I, J)** Western blot was used to detect the expression of PR-A, LC3II/I, Beclin 1, p62 and cytochrome C in ovarian tissues; **(K, L)** The MMP level and COX4 expression in granulosa cells was detected by JC-1 method and RT-qPCR; **(M)** Mitochondrial damage in ovarian tissue was observed by electron microscopy (green arrow marked the damaged mitochondria, red arrow marked autophagosomes). *P<0.05 vs DHEA-induced PCOS-FMT. All data showed as mean ± SD.

There was no significant change of body weight between DHEA-induced PCOS-FMT group and E. coli Nissle 1917-FMT group (**Figure 5A**). HE observation showed that the characteristic polycystic ovary morphology was found in the DHEA-induced PCOS-FMT group, with less corpus luteum, thinner granulosa cell layer, increased number of antral follicles and fewer primordial follicles (**Figure 5B**). E. coli Nissle 1917-FMT intervention alleviated PCOS symptoms (**Figure 5B**). Compared with the DHEA-induced PCOS-FMT group, the glucose level has no change, while the INS level was clearly reduced in the E. coli Nissle 1917-FMT group (**Figure 5C**). Compared with the DHEA-induced PCOS-FMT group, the levels of FSH, P and IL-22 were increased, and the LH and Testo levels were decreased in the E. coli Nissle 1917-FMT group (**Figures 5E–H**). E. coli Nissle 1917-FMT intervention increased the expression of p62 and PR-A, and decreased the expression of LC3II/I, Beclin 1 and cytochrome C in ovarian tissues of PCOS mice (**Figures 5I, J**). In addition, the expression of COX4 and MMP level in granulosa cells were increased after E. coli Nissle 1917-FMT intervention (**Figures 5K, L**). Electron microscopy showed that the damaged mitochondria and autophagosomes in the ovarian tissues of mice was reduced after E. coli Nissle 1917-FMT intervention (**Figure 5M**). These results demonstrated that E. coli Nissle 1917-FMT ameliorated mitochondrial damage in granulosa cells of PCOS mice.

### 3.6 IL-22 mediated mitochondrial damage in granulosa cells of PCOS mice ameliorated by EcN

**Figure 6:**
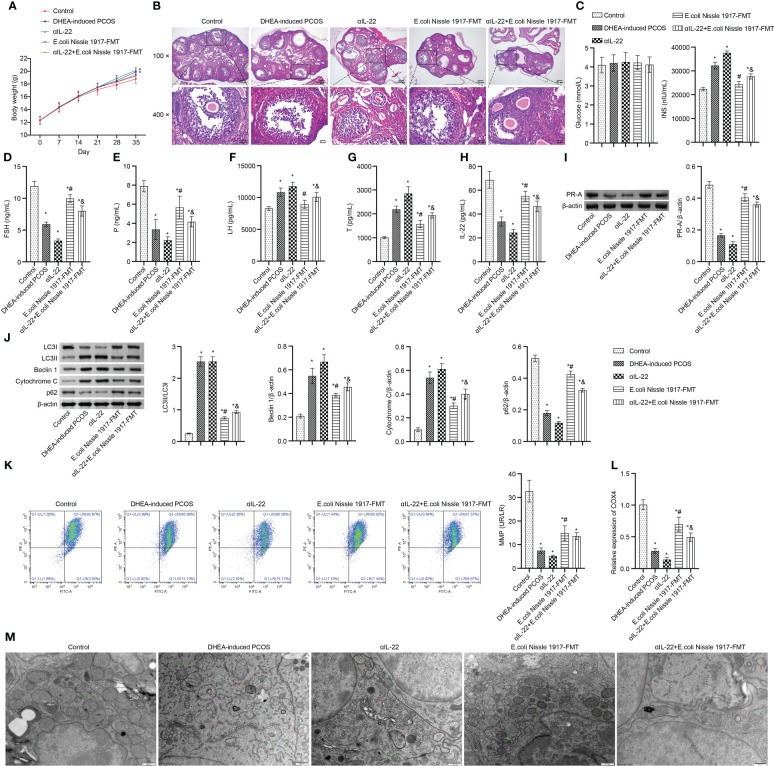
IL-22 mediated EcN ameliorated mitochondrial damage in PCOS mice. **(A)** Body weight; **(B)** HE staining; **(C)** Glucose and INS levels; **(D–H)** Serum levels of FSH, P, LH, Testo and IL-22 were detected by Elisa; **(I, J)** Western blot was used to detect the expression of PR-A, LC3II/I, Beclin 1, p62 and cytochrome C in ovarian tissues; **(K, L)** The MMP level and COX4 expression in granulosa cells was detected by JC-1 method and RT-qPCR; **(M)** Mitochondrial damage in ovarian tissue was observed by electron microscopy (green arrow marked the damaged mitochondria, red arrow marked autophagosomes). *P<0.05 vs Control, #*P*<0.05 vs DHEA-induced PCOS, &*P*<0.05. All data showed as mean ± SD.

Body weight in PCOS mice was higher than that in Control group, which was up-regulated by the αIL-22 intervention, down-regulated by the E. coli Nissle 1917-FMT and αIL-22+E. coli Nissle 1917-FMT intervention (**Figure 6A**). E. coli Nissle 1917-FMT inhibited the action of αIL-22 in PCOS mice (**Figure 6A**). HE observation showed that ovaries in the Control group contained follicles at different stages of development and several corpus luteum (**Figure 6B**). The characteristic polycystic ovary morphology was found in the ovaries of mice in the DHEA-induced PCOS group, with less corpus luteum, thinner granulosa cell layer, increased number of antral follicles and fewer primordial follicles (**Figure 6B**). αIL-22 exacerbated PCOS symptoms (**Figure 6B**). E. coli Nissle 1917-FMT intervention alleviated PCOS symptoms and partially reversed the effect of αIL-22 on PCOS mice (**Figure 6B**). There was no significant change of glucose level in different groups (**Figure 6C**). αIL-22 promoted the INS level in PCOS mice, which was down-regulated by E. coli Nissle 1917-FMT intervention (**Figure 6C**). Compared with the DHEA-induced PCOS group, the serum FSH, P and IL-22 levels were decreased, and the LH and T levels were increased in the αIL-22 group (**Figures 6D–H**). Compared with αIL-22 group, serum FSH, P and IL-22 levels were significantly increased, and the LH and T levels were significantly decreased in αIL-22 + E. coli Nissle 1917-FMT group (**Figures 6D–H**). αIL-22 increased the expression of LC3II/I, Beclin 1 and cytochrome C, while decreased the expression of p62 and PR-A in ovarian tissues of PCOS mice (**Figures 6I, J**). Compared with the αIL-22 group, the expression of LC3II/I, Beclin 1 and cytochrome C in ovarian tissues of mice were decreased, and the expression of p62 and PR-A was increased in αIL-22 + E. coli Nissle 1917-FMT group (**Figures 6I, J**). αIL-22 decreased MMP level and COX4 expression, and increased the damaged mitochondria and autophagosomes in ovarian granulosa cells of PCOS mice, which was reversed by E. coli Nissle 1917-FMT intervention (**Figures 6K–M**). These results demonstrated that IL-22 mediated mitochondrial damage in granulosa cells of PCOS mice improved by E. coli Nissle 1917-FMT.

### 3.7 IL-22 was involved in the process of PCOS disease

**Figure 7:**
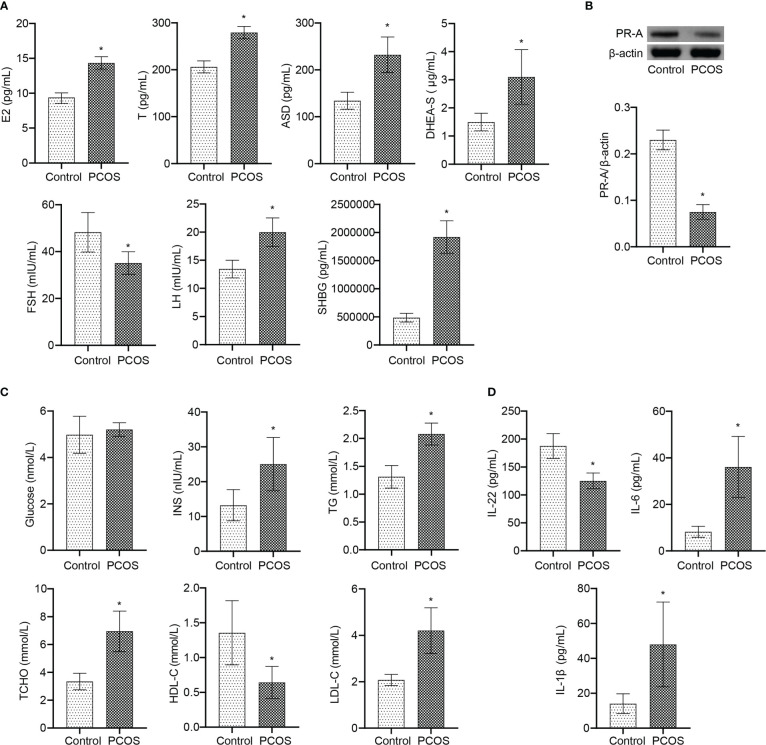
IL-22 was involved in the occurrence and treatment of PCOS. **(A)** Serum E2, T, ASD, DHEA-S, FSH, LH, and SHBG levels were detected by ELISA; **(B)** The PR-A expression was detected by western blot; **(C)** The levels of Glucose, INS, TG, T-CHO, HDL-C and LDL-C were detected by biochemical kit; **(D)** Serum levels of IL-22, IL-6 and IL-1β were analyzed by ELISA. *P<0.05 vs Control. All data showed as mean ± SD.

Further analysis at the clinical level showed that there was no significant changes of age and BMI between Control and PCOS groups (**Table 1**). Serum FSH level in PCOS patients was lower than Control group (**Figure 7A**). The levels of E2, T, ASD, DHEA-S, LH, and SHBG were increased in PCOS patients (**Figure 7A**). The PR-A protein expression was down-regulated in PCOS patients (**Figure 7B**). In addition, patients with PCOS had the decrease of HDL-C level, and the increase of INS, TG, T-CHO, and LDL-C levels, while no significant changes in blood glucose level (**Figure 7C**). The serum levels of IL-6 and IL-1β increased, and IL-22 decreased in PCOS patients (**Figure 7D**). These results demonstrated that PCOS patients were associated with the decrease of IL-22 level, PR-A level, and mitochondrial damage in granulosa cells, which was the therapeutic target of E. coli Nissle 1917.

## 4 Discussions

High-dose *B. coagulans* (6.50 mg/kg) can cooperate with genitin and daidzein in improving hormone, oxidative stress, menstrual cycle, and ovarian physiology in PCOS rats (29). Prebiotics (inulin) and metformin alleviate PCOS by anti-inflammatory and modulating the gut microbiota, involving an increase in *Bifidobacterium* and a decrease in *Proteus*, *Helicobacter*, and *Paracinobacterium* (30). Synbiotics (including IBRC-M10785, IBRC-M10783, and IBRC-M10771+ inulin) supplementation for 12 weeks had beneficial effects on insulin resistance markers, triglyceride, VLDL-cholesterol concentrations, and plasma atherosclerosis index in PCOS women (31). Progesterone is a key regulator of normal uterine function, and the expression, regulation and signaling pathways of progesterone receptor (PR) in the nucleus are associated with progesterone resistance in women with PCOS (32). Our study found that EcN promoted the recovery of sex hormone levels and ovarian tissue morphology, promoted the expression of IL-22, COX4 and PR-A in granulosa cells, and inhibited mitophagy in PCOS mice. All these proved that EcN was a beneficial probiotic in DHEA-induced PCOS mice, which was helpful to clinical application.

The improvement of PCOS in rats by Bushen-Huayu decoction was related to the increase of α diversity of intestinal flora, the increase of *Lactobacillus*, *Allobaculum*, *Ruminococcaceae_UCG-014* and *Bacteroides*, and the promotion of carbohydrate and protein metabolism (33). Decreased IL-22 gene expression and *Allobaculum* depletion were observed in the ileum of NOD2-deficient mice fed HFD, which may be related to an imbalance in the Th17/Th1 cell population (34). In overweight and obese women with PCOS, probiotic supplements had no additional beneficial effects on anthropometric parameters, carbohydrate metabolism, or androgen status-only reduced diet (35). EcN decreased the number of gut microbiota, and significantly increased the abundance of *Adlercreutzia*, *Allobaculum*, *Escherichia-Shigella* and *Ileibacterium* in PCOS mice. FMT also proved that EcN improved PCOS through gut microbiota. However, the effect on overweight or obese PCOS mice remains unknown. These results demonstrated that EcN could improve the disease representation and mitochondrial damage in PCOS mice, but the effect in overweight or obese PCOS mice still needs to be further studied.

As many as 60 genes encoding putative carbohydrate active enzymes (CAZymes) have been identified in the genome of *Allobaculum. mucolyticum*, whose CAZyme secretion may promote bacterial colonization and degradation of the mucus layer (36). Corn silk can improve the *Allobaculum*, *Turicibacter*, *Romboutsia*, *Streptococcus*, *Sporobacter*, *Christensenella*, *ClostridiumXVIII* and *Rikenella* abundance, as well as bile acid metabolism (CDCA, LCA, etc.) in high-fat diet mice and play an anti-hypercholesterolemia effect (37). Goose deoxycholic acid (CDCA) can activate intestinal farnesol X receptor and improve glucose metabolism in patients with PCOS (38). Our metabolomics analysis showed that EcN has the most significant influence on amino sugar and nucleotide sugar metabolism of gut microbiota in PCOS mice. IL-22 was positively associated with *Ileibacterium*, *Adlercreutzia* and Progesterone, negatively associated with RF39, Luteinizing hormone, Testosterone, N−Acetylglucosamin, L−Fucose and N−Acetylmannosamin. These results suggested that the change of IL-22 levels might be a potential intervention node for EcN to improve PCOS mice.

PCOS patients exhibit progesterone resistance, indicating the role of progesterone receptors (PR) in disease etiology and prognosis (39). Vitamin A supplementation increased jejunal IL-22 expression and jejunal *Akkermansia*, *uncultured-Muribaculaceae*, *Allobaculum*, *Lachnospiraceae NK4A136 group*, *Rummeliibacillus* and *Parasutterella* abundance (40). Clinical trials have further confirmed the decrease of IL-22 and PR-A in PCOS patients. The significant reduction of PR-A gene in follicular granulosa cells in women with PCOS may be considered as a marker of defective granulosa cell maturation or follicular stagnation (41). In addition, *Adlercreutzia* was positively associated with IL-22 and Progesterone, negatively associated with Luteinizing hormone and Testosterone. Therefore, we hypothesized that *Adlercreutzia* may be a common microbe of EcN to improve the levels of IL-22 and cholesterol in peripheral blood of PCOS mice.

## 5 Conclusions

In conclusion, our study demonstrated that IL-22 was involved in the process of patients with PCOS, and was inversely associated with the disease status of PCOS. FMT and IL-22 inhibitor intervention again revealed that EcN could improve intestinal microbial diversity, amino sugar and nucleotide sugar metabolism, ovarian tissue damage and mitochondrial dysfunction in DHEA-induced PCOS mice, but this process was mediated by IL-22 expression. *Adlercreutzia* was positively correlated with IL-22 expression and may be a biomarker for EcN treatment of PCOS.

## 6 Limitations of study

In this study, we determined that EcN improve PCOS disease characterization in the DHEA-induced PCOS mice through gut microbiota and IL-22. We also found that *Adlercreutzia* was positively correlated with IL-22 expression in EcN treatment. But we did not perform the pregnancy experiment to verify the treatment effect of EcN in PCOS mice, which was a limitation. The interaction between *Adlercreutzia* and IL-22 was unknown. These were worth to explore in IL-22 gene knockout mouse model. At last, we did not collect the enough intervention data from non-PCOS-IVF and PCOS-IVF patients, which might be a limitation in our study. These will be points that we need to explore or expand in the future.

## Data availability statement

The data presented in the study are deposited in the NCBI BioProject repository, accession number PRJNA930556.

## Ethics statement

The studies involving human participants were reviewed and approved by Ethics Committee of Hunan Provincial Maternal and Child Health Hospital (2022037). The patients/participants provided their written informed consent to participate in this study. The animal study was reviewed and approved by Ethics Committee of Hunan Provincial Maternal and Child Health Hospital (2022037).

## Author contributions

Conceptualization: ML; Methodology: YC; Data curation: ML, YC, XP, and HC; Formal analysis: ML, YC, and LF; Visualization: ML, YC, and YW; Funding acquisition: ML; Writing-original draft: ML and YC. All authors agree to be accountable for the content of the work. All authors contributed to the article and approved the submitted version.

## Supplementary material

Supplementary Figure 1 The experimental flow chart.

Supplementary Figure 2 The representative picture of the vaginal smears in Control group and DHEA-induced PCOS group (1-7 days).

Supplementary Figure 3 The representative picture of the vaginal smears in Control group and DHEA-induced PCOS group (8-14 days).
