# Supplementary figures and images for: E. coli Nissle 1917 ameliorates mitochondrial injury of granulosa cells in polycystic ovary syndrome through promoting gut immune factor IL-22 via gut microbiota and microbial metabolism
Source: Front Immunol. 2023 May 19;14:1137089. doi: 10.3389/fimmu.2023.1137089 (PMC10235540; doi:10.3389/fimmu.2023.1137089)

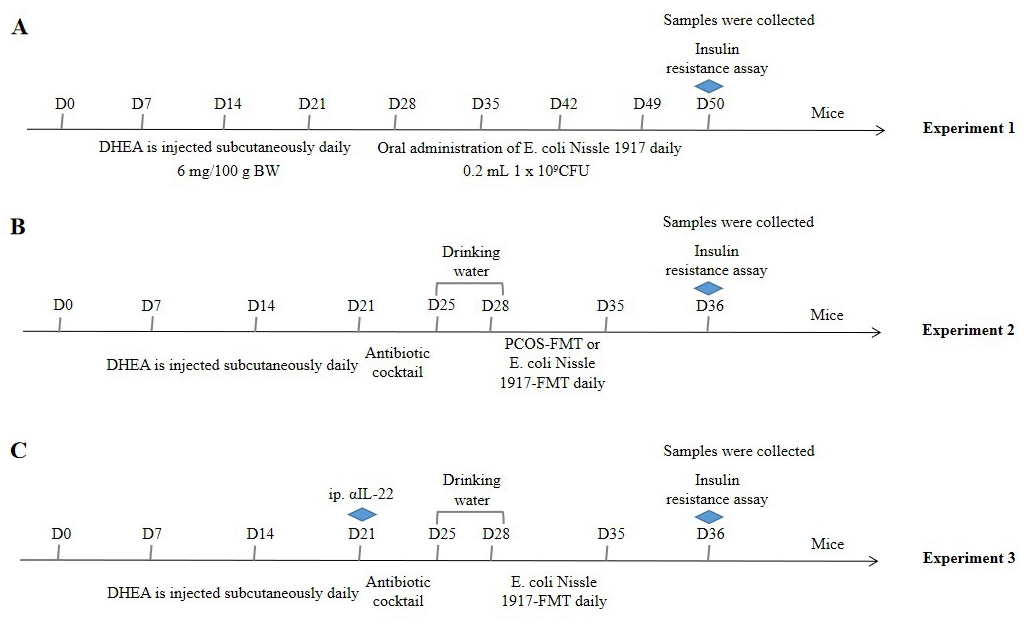

Supplement: Supplementary Figure 1 — The experimental flow chart. [file Image_1.jpeg]

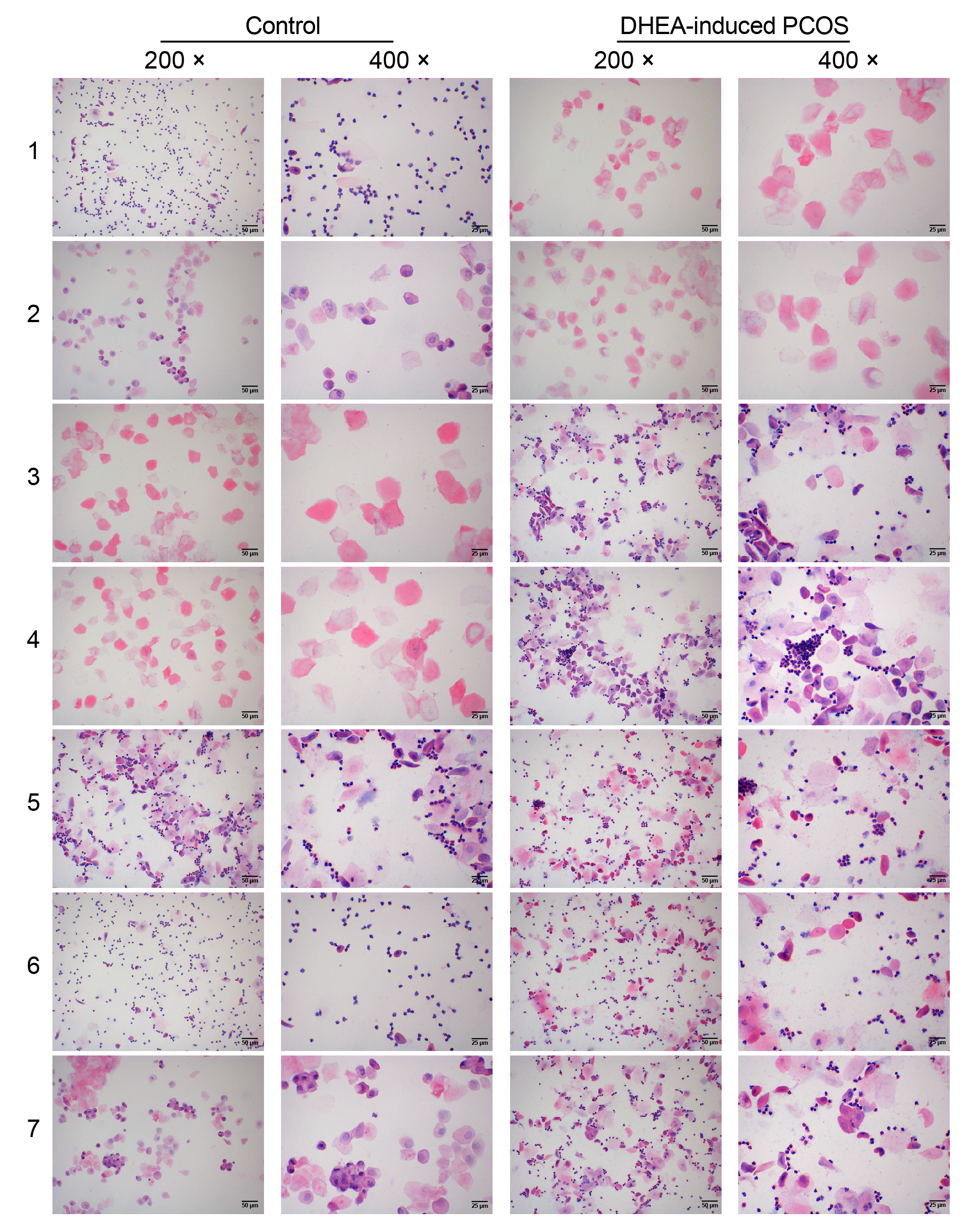

Supplement: Supplementary Figure 2 — The representative picture of the vaginal smears in Control group and DHEA-induced PCOS group (1-7 days). [file Image_2.jpeg]

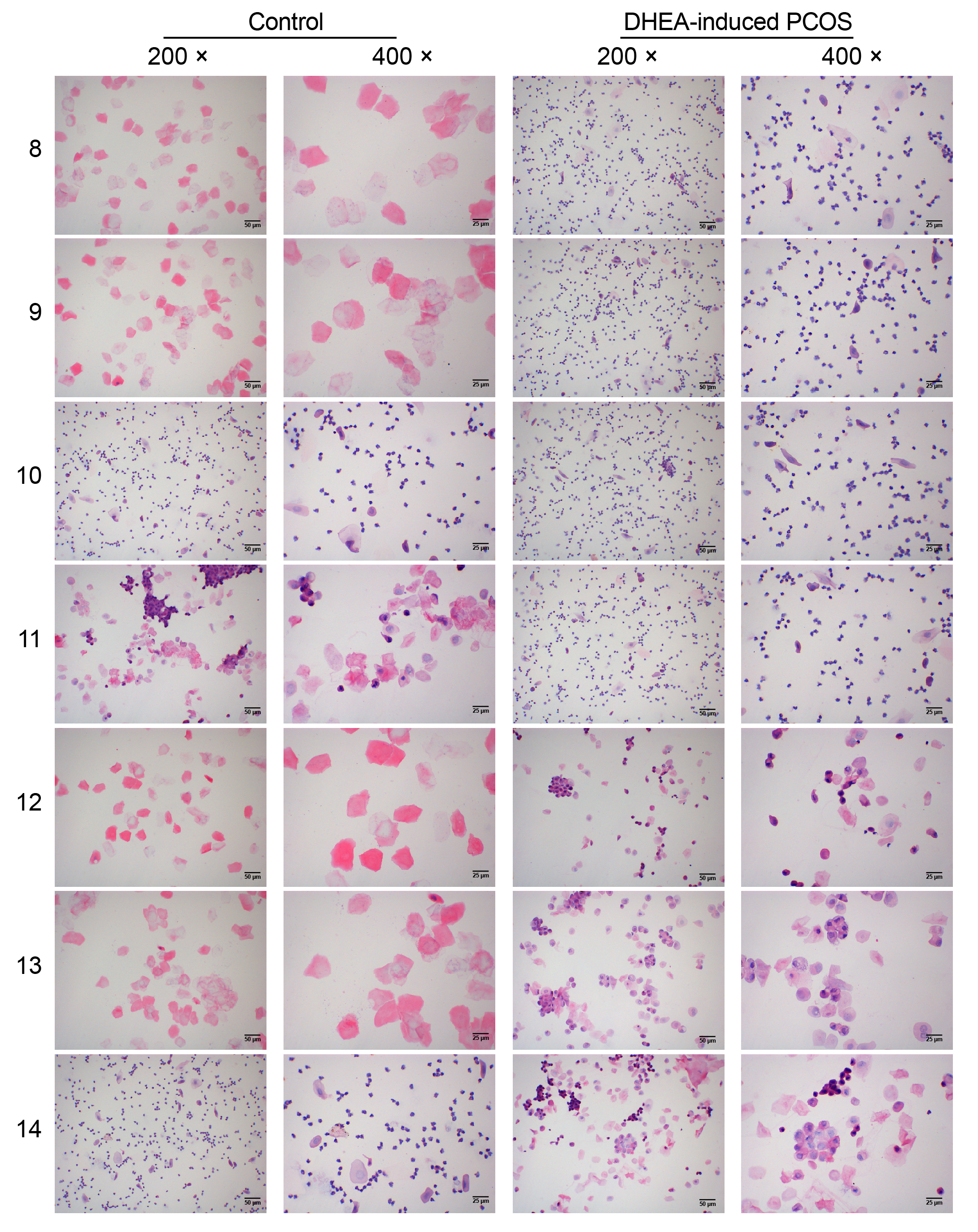

Supplement: Supplementary Figure 3 — The representative picture of the vaginal smears in Control group and DHEA-induced PCOS group (8-14 days). [file Image_3.jpeg]
